# Working-age mortality is still an important driver of stagnating life expectancy in the United States

**DOI:** 10.1073/pnas.2318276121

**Published:** 2024-01-16

**Authors:** Antonino Polizzi, Jennifer Beam Dowd

**Affiliations:** ^a^Department of Sociology, University of Oxford, Oxford OX1 1JD, United Kingdom; ^b^Leverhulme Centre for Demographic Science, University of Oxford, Oxford OX1 1JD, United Kingdom; ^c^Nuffield College, University of Oxford, Oxford OX1 1NF, United Kingdom; ^d^Nuffield Department of Population Health, University of Oxford, Oxford OX3 7LF, United Kingdom

Abrams et al. (1) ask what life expectancy would have been in the United States (US) if age-specific mortality trends in 2000 to 2009 had continued through 2010 to 2019. They estimate higher-than-observed remaining life expectancy at age 25 (e_25_) in this counterfactual scenario (1.2 and 2.1 y for females and males, respectively, in 2019). Abrams et al. find that most of the counterfactual improvements in life expectancy come from older ages (65+), contrary to explanations that emphasize working-age (25 to 64) mortality as the largest contributor to the recent US life expectancy stagnation.

This counterfactual approach provides a welcome new perspective on US life expectancy trends. Nonetheless, the chosen counterfactual provides only one possible lens on US life expectancy post-2009, one that may overemphasize the contribution of older-age mortality. To illustrate, Fig. 1 shows annual rates of mortality improvement (ROMI) over the two decades 2000 to 2009 (bars) and 2010 to 2019 (lines) for the US and four other high-income countries (2). Abrams et al. use the US ROMI from 2000 to 2009 for their counterfactual calculations (leftmost column). Older-age mortality was improving substantially in that decade, whereas working-age mortality had already stagnated. We note that looking internationally, (a) a slowdown in older-age mortality improvements was common in 2010 to 2019, even if it was generally larger in the United States; (b) stagnating or increased mortality in working ages was uncommon, except for the United Kingdom (3).

**Fig. 1.**
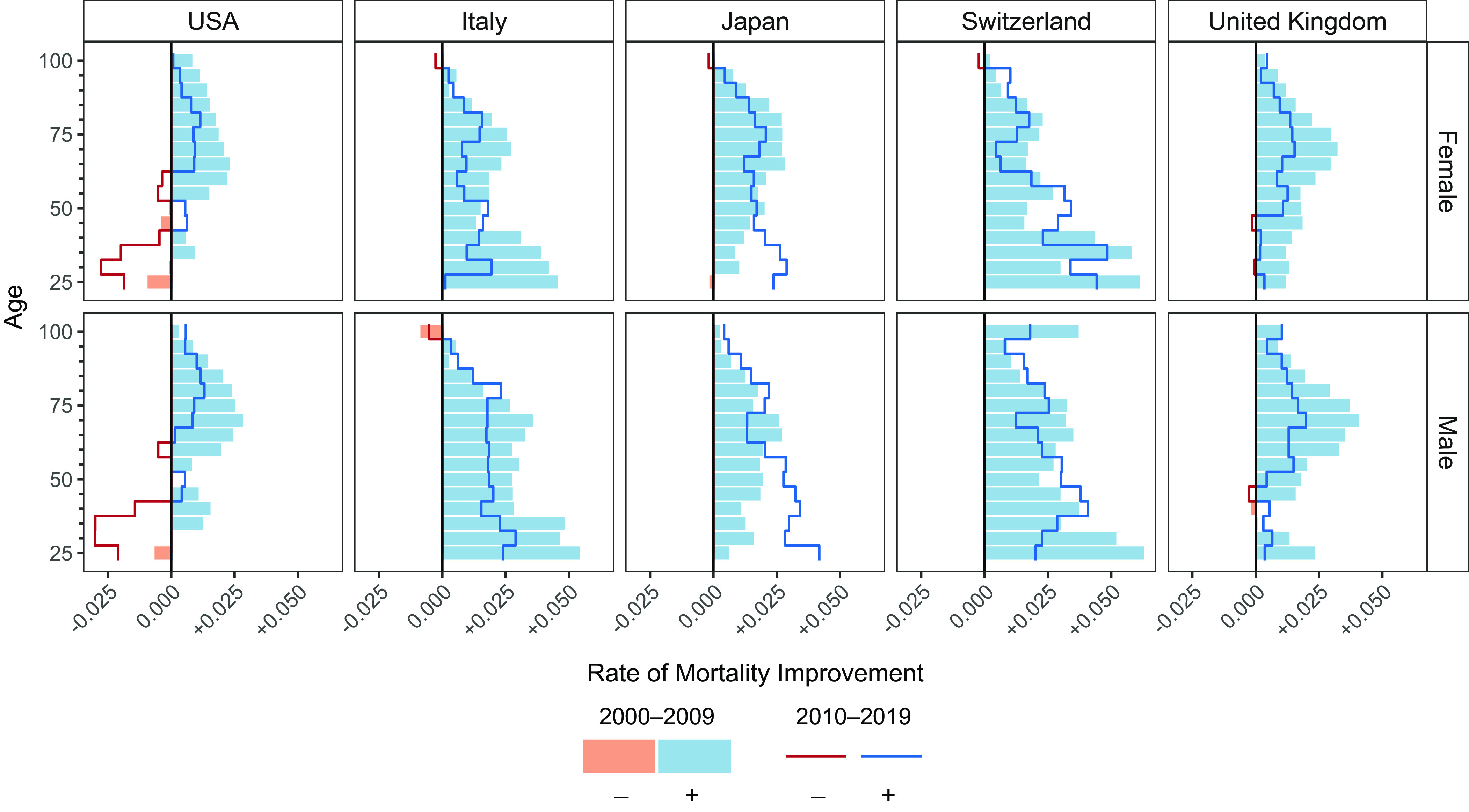
ROMI by 5-y age groups in the United States (USA) and four other high-income countries, 2000 to 2009 and 2010 to 2019. Source: Authors’ calculation based on data from the Human Mortality Database (4).

Thus, the counterfactual estimates of Abrams et al. reflect slowing improvements in older-age mortality no longer compensating for stagnating working-age mortality in the United States. We ask a different counterfactual question, visualized in Fig. 2: What would US e_25_ have been if mortality trends after 2009 had looked similar to those in other high-income countries? Each country panel shows US e_25_ after applying the country-specific 2010 to 2019 ROMI, separating the contributions of working and older ages. The USA panel replicates Abrams et al.’s counterfactual of continuing the 2000 to 2009 mortality trends. Contrary to the within-US results, most scenarios show that ages 25 to 64 contribute most to e_25_ differences. This holds in 16 (female) and 28 (male) out of 32 possible counterfactuals with 2010 to 2019 ROMI from Human Mortality Database countries (4). In some cases, improving only working-age mortality at the 2010 to 2019 pace of other countries would have affected e_25_ similarly to continuing all-age mortality trends at the US pace of 2000 to 2009.

**Fig. 2.**
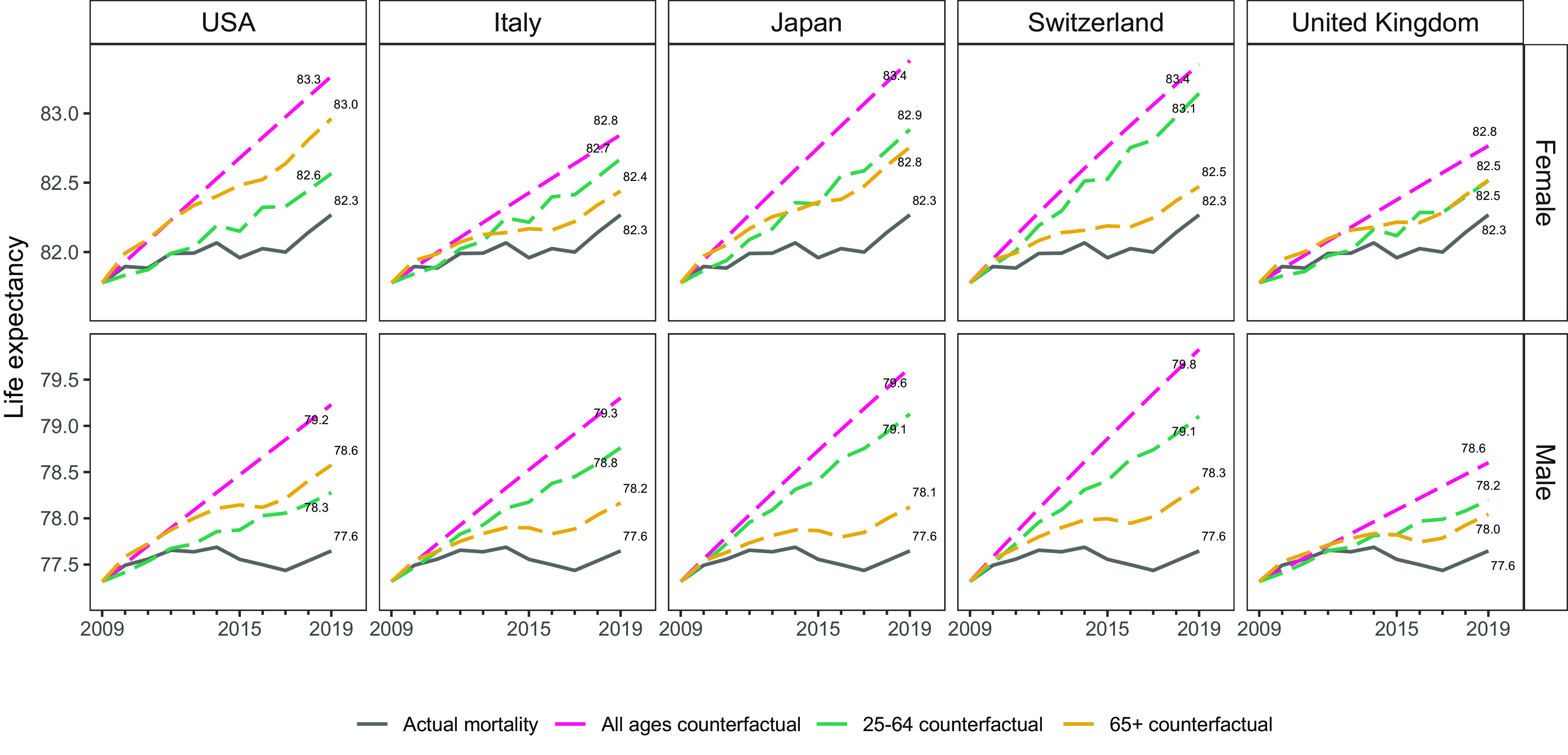
Observed and counterfactual life expectancy at age 25 in the United States, 2009 to 2019 (e_25_ + 25). Note: The USA panel assumes continuation of US 2000 to 2009 ROMI. Remaining panels assume country-specific 2010 to 2019 ROMI would have applied in the United States. Source: Authors’ calculation based on data from the Human Mortality Database (4).

The estimates of Abrams et al. reflect a comparatively unusual counterfactual scenario where working-age mortality was already stagnating, which accentuates the contributions of slowing older-age mortality improvements. These slowdowns are more pronounced than in other high-income countries and merit better understanding, as Abrams et al. suggest. However, in the bigger picture of lagging US life expectancy globally (5), rising working-age mortality may still play a more important role. Counterfactual analysis, and its potential to consider multiple diverse scenarios, will continue to be an indispensable tool to address this central demographic question (6–8).

## Data Availability

All data used in the analysis are publicly available, under a Creative Commons Attribution 4.0 International License, from the Human Mortality Database: https://mortality.org (4). To facilitate replication, all data and analysis files have been deposited in an Open Science Framework (OSF) repository: https://doi.org/10.17605/osf.io/vjkux (9).
